# Supplementary material for: Upcycling of Waste Cherries Produces an Anthocyanin-Rich Powder That Protects Against Amyloid-β Toxicity in C. elegans
Source: Antioxidants (Basel). 2025 Aug 13;14(8):995. doi: 10.3390/antiox14080995 (PMC12382708; doi:10.3390/antiox14080995)
Supplement: Supplementary file 1 [file antioxidants-14-00995-s001.zip › antioxidants-3689171-supplementary.pdf]

Supplementary Materials

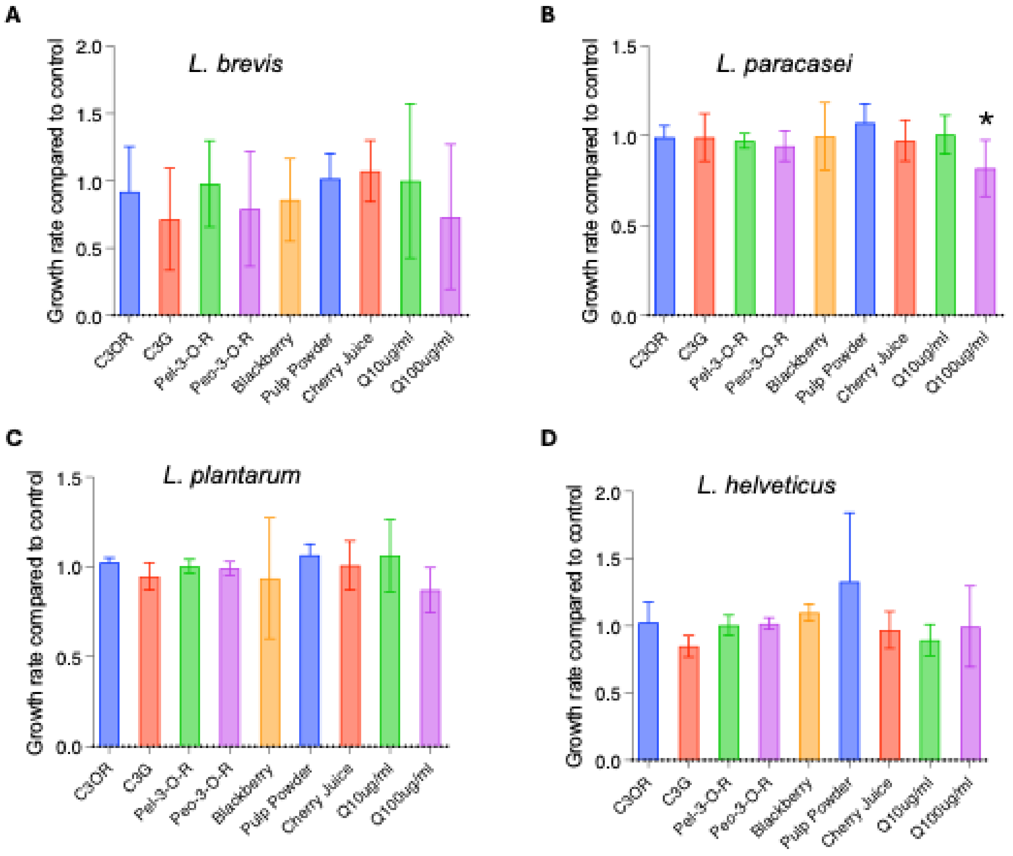

Supplementary Figure S1: Bacterial growth rates

A-D) Maximum growth rates of probiotic bacteria. Individual anthocyanins at 50 μg/mL, or cherry products themselves have no effects on growth rates of *L. brevis*, *L. paracasei*, *L. plantarum* and *L. helveticus.* * $p < 0.05$, Student's Test, Two-tailed, homoscedastic. Error bars represent ± SEM. Combined results from 5-10 biological replicates shown.

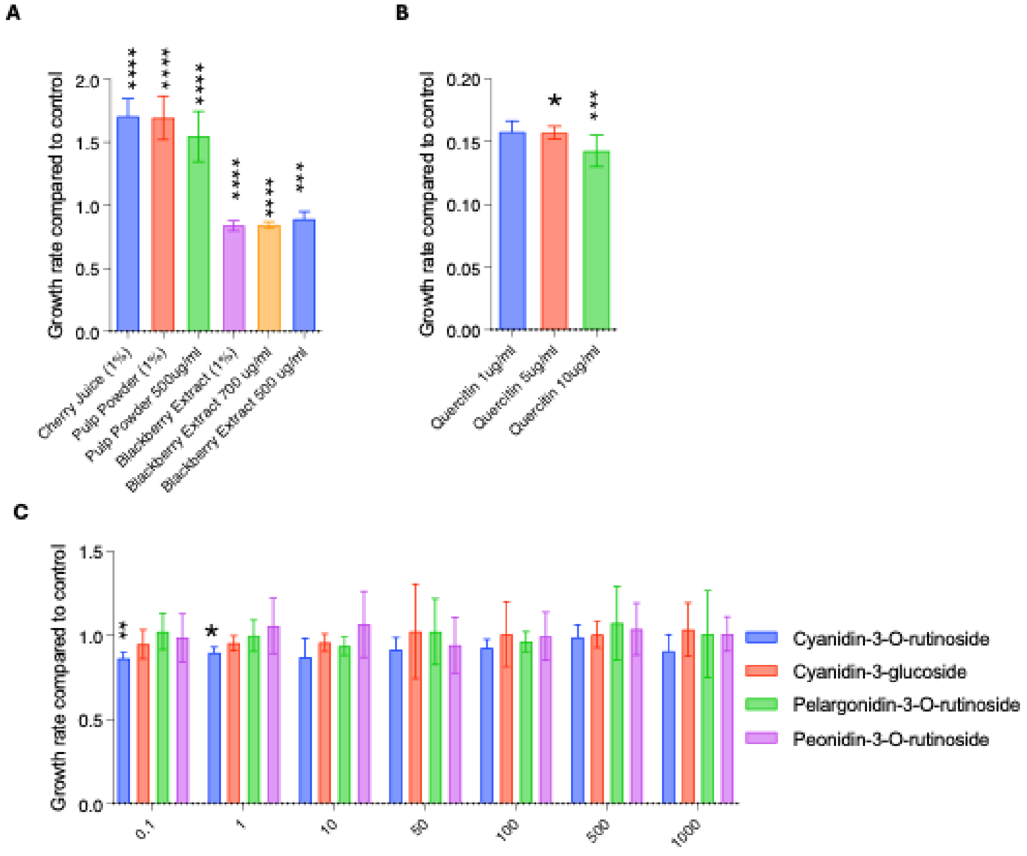

Supplementary Figure S2: Effect of cherry products and anthocyanins on OP50 growth.

A-C) Maximum growth rates of OP50 E. coli. Individual anthocyanins provided at 100 μg/mL, Concentrations of cherry products in Table S1.) * $p < 0.05$; ** $p < 0.01$; ****, $p < 0.0001$, Student's Test, Two-tailed, homoscedastic. Error bars represent ± SEM. Combined results from 5-10 biological replicates shown.

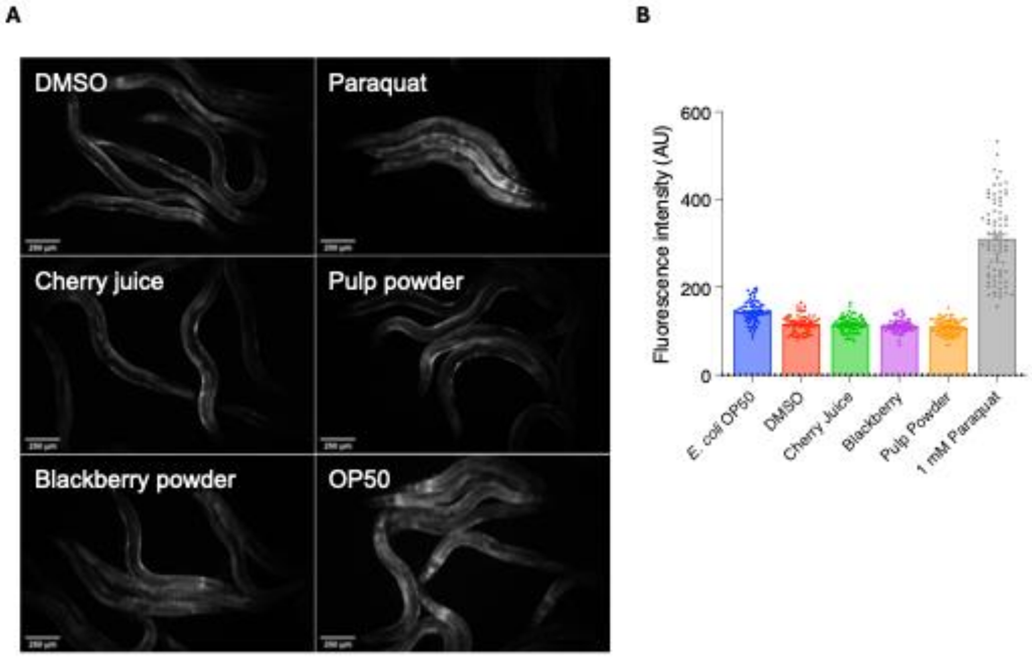

Supplementary Figure S3: Effect of cherry products on Nrf2/SKN-1 activation reporter *gst-4::GFP*.

A) Representative fluorescence images of *C. elegans* expressing *gst-4::GFP*. B) Mean fluorescence intensity of *C. elegans* expressing *gst-4::GFP*. * $p < 0.05$; ** $p < 0.01$; ****, $p < 0.0001$, one-way Brown-Forsythe ANOVA. Error bars represent ± SEM. Individual data points showed as dots. Combined results from 4 biological replicates shown.
